# Long-term surgical outcomes of bile duct tumor thrombus versus portal vein tumor thrombus for hepatocellular carcinoma: a propensity score matching analysis

**DOI:** 10.3389/fonc.2024.1372123

**Published:** 2024-04-02

**Authors:** Yi-Nan Li, Shao-Ming Wei, Yang-Kai Fu, Zhen-Xin Zeng, Li-Ming Huang, Jia-Hui Lv, Wei-Zhao Chen, Yong-Gang Wei, Zhi-Bo Zhang, Jian-Yin Zhou, Jia-Yi Wu, Mao-Lin Yan

**Affiliations:** ^1^ Shengli Clinical Medical College, Fujian Medical University, Fuzhou, Fujian, China; ^2^ Department of Hepatobiliary Pancreatic Surgery, Fujian Provincial Hospital, Fuzhou, Fujian, China; ^3^ Department of Liver Surgery, Liver Transplantation Center, West China Hospital of Sichuan University, Chengdu, Sichuan, China; ^4^ Department of Hepatobiliary Pancreatic Surgery, First Affiliated Hospital of Fujian Medical University, Fuzhou, Fujian, China; ^5^ Department of Hepatobiliary Surgery, Zhongshan Hospital of Xiamen University, Xiamen, Fujian, China

**Keywords:** hepatocellular carcinoma, macroscopic bile duct tumor thrombus, macroscopic portal vein tumor thrombus, overall survival, propensity score matching analysis

## Abstract

**Background:**

Portal vein tumor thrombus (PVTT) seriously affects the prognosis of hepatocellular carcinoma (HCC). However, whether bile duct tumor thrombus (BDTT) significantly affects the prognosis of HCC as much as PVTT remains unclear. We aimed to compare the long-term surgical outcomes of HCC with macroscopic PVTT (macro-PVTT) and macroscopic BDTT (macro-BDTT).

**Methods:**

The data of HCC patients with macro-BDTT or macro-PVTT who underwent hemihepatectomy were retrospectively reviewed. A propensity score matching (PSM) analysis was performed to reduce the baseline imbalance. The recurrence-free survival (RFS) and overall survival (OS) rates were compared between the cohorts.

**Results:**

Before PSM, the PVTT group had worse RFS and OS rates than the BDTT group (P = 0.043 and P = 0.008, respectively). Multivariate analyses identified PVTT (hazard ratio [HR] = 1.835, P = 0.016) and large HCC (HR = 1.553, P = 0.039) as independent risk factors for poor OS and RFS, respectively. After PSM, the PVTT group had worse RFS and OS rates than the BDTT group (P = 0.037 and P = 0.004, respectively). The 3- and 5-year OS rates were significantly higher in the BDTT group (59.5% and 52.1%, respectively) than in the PVTT group (33.3% and 20.2%, respectively).

**Conclusion:**

Aggressive hemihepatectomy provides an acceptable prognosis for HCC patients with macro-BDTT. Furthermore, the long-term surgical outcomes of HCC patients with macro-BDTT were significantly better than those of HCC patients with macro-PVTT.

## 1 Introduction

Hepatocellular carcinoma (HCC), one of the most common malignant neoplasms, ranks sixth in morbidity and third in mortality among malignant tumors worldwide (1). As both the portal vein and bile duct are surrounded by the Glisson sheath, HCC has a propensity to invade the portal vein and its branches to form portal vein tumor thrombus (PVTT) or spread into the bile duct to form bile duct tumor thrombus (BDTT) (2, 3).

PVTT seriously affects the prognosis of HCC. According to the Barcelona Clinic for Liver Cancer Staging System (4), macroscopic PVTT (macro-PVTT) is considered indicative of an advanced stage of HCC, and surgical intervention is not recommended due to the poor prognosis (5). Therefore, systemic therapy is the only suggested treatment for such patients. In Asian countries, such as China and Japan, more aggressive anticancer therapy, including surgery, is recommended for selected HCC patients with macro-PVTT (6–8). Previous studies (7, 9, 10) have revealed that an aggressive surgery considerably enhances the prognosis in HCC patients with tumor thrombus beyond the main trunk portal vein as compared to non-surgical procedures, with a 5-year overall survival (OS) of 12%–33% after surgery (9, 11).

Historically, guidelines did not categorize BDTT as a factor for staging purposes due to its rarity and the controversy over its impact on long-term prognosis (12, 13). Several studies reported that BDTT severely affects the prognosis of HCC patients (14–16). However, other studies demonstrated that the presence of BDTT did not affect the OS of HCC patients (17, 18). A meta-analysis proposed that HCC with BDTT had worse histologic features, higher rates of macrovascular and lymphovascular invasion, and poorer differentiation than HCC without BDTT, and that there was no difference in 1- and 3-year OS rates after hepatectomy between the two groups, but the 5-year OS rate was worse in the BDTT group (19). Moreover, previous literature (20, 21) reported a 5-year OS of approximately 6.7–28% for HCC with BDTT, and surgery was not considered a viable option for HCC patients with macroscopic BDTT (macro-BDTT). However, some recent studies (18, 22–24) have concluded that curative hemihepatectomy improves long-term outcomes in HCC patients with macro-BDTT, with a 5-year postoperative survival rate of 44.2%–52.8% (23, 24).

Overall, aggressive hemihepatectomy can improve the prognosis of selected HCC patients with macro-PVTT and macro-BDTT. However, differences in prognosis between HCC patients with macro-PVTT and those with macro-BDTT after surgery have been rarely reported in the literature. Only one study (25) compared the prognosis of the two types of patients and its results indicated that HCC with BDTT had a worse prognosis than HCC with PVTT. Nonetheless, this finding contradicted our clinical experience. Currently, whether macro-BDTT significantly affects the prognosis as much as macro-PVTT remains speculative and controversial.

This study aimed to compare the outcomes of HCC in patients with macro-PVTT and macro-BDTT after surgery using propensity score matching (PSM) and to identify the risk factors that influence overall survival and recurrence-free survival.

## 2 Methods

### 2.1 Patients

This retrospective study enrolled HCC patients with macro-BDTT or macro-PVTT who had undergone curative hemihepatectomy between September 2014 and July 2018 at four major cancer institutions: the Fujian Provincial Hospital (Fuzhou, China), West China Hospital of Sichuan University (Chengdu, China), First Affiliated Hospital of Fujian Medical University (Fuzhou, China), and Zhongshan Hospital of Xiamen University (Xiamen, China). The study protocol was approved by the Institutional Review Board of Fujian Provincial Hospital (approval number: K2017-058-04). Written informed consent was obtained from all participants or their legal guardians. Clinical and pathological data were retrospectively obtained from a prospectively maintained database.

All the patient images and specimens were separately reviewed by two experienced radiologists and two experienced pathologists in each participating hospital, and the diagnosis of HCC with macro-BDTT or macro-PVTT was confirmed by preoperative imaging and histopathological examination. Macro-BDTT was classified as B3 BDTT, indicating that the tumor thrombus invaded the initial branches of the bile duct, and B4 BDTT, indicating the invasion of the common hepatic duct (26). Macro-PVTT diagnosis indicated that the tumor thrombus was in the main trunk and the first branches of the portal vein (27). However, patients with PVTT involving the main trunk were excluded owing to a lack of recommendations for surgery (9, 10). In addition, the criteria for curative resection were as follows: the margin was free, as shown by histology, and the serum alpha-fetoprotein (AFP) level and postoperative radiographic examinations, including magnetic resonance imaging (MRI) and computed tomography (CT), showed no signs of tumor 3 months postoperatively (28–30).

**Figure 1:**
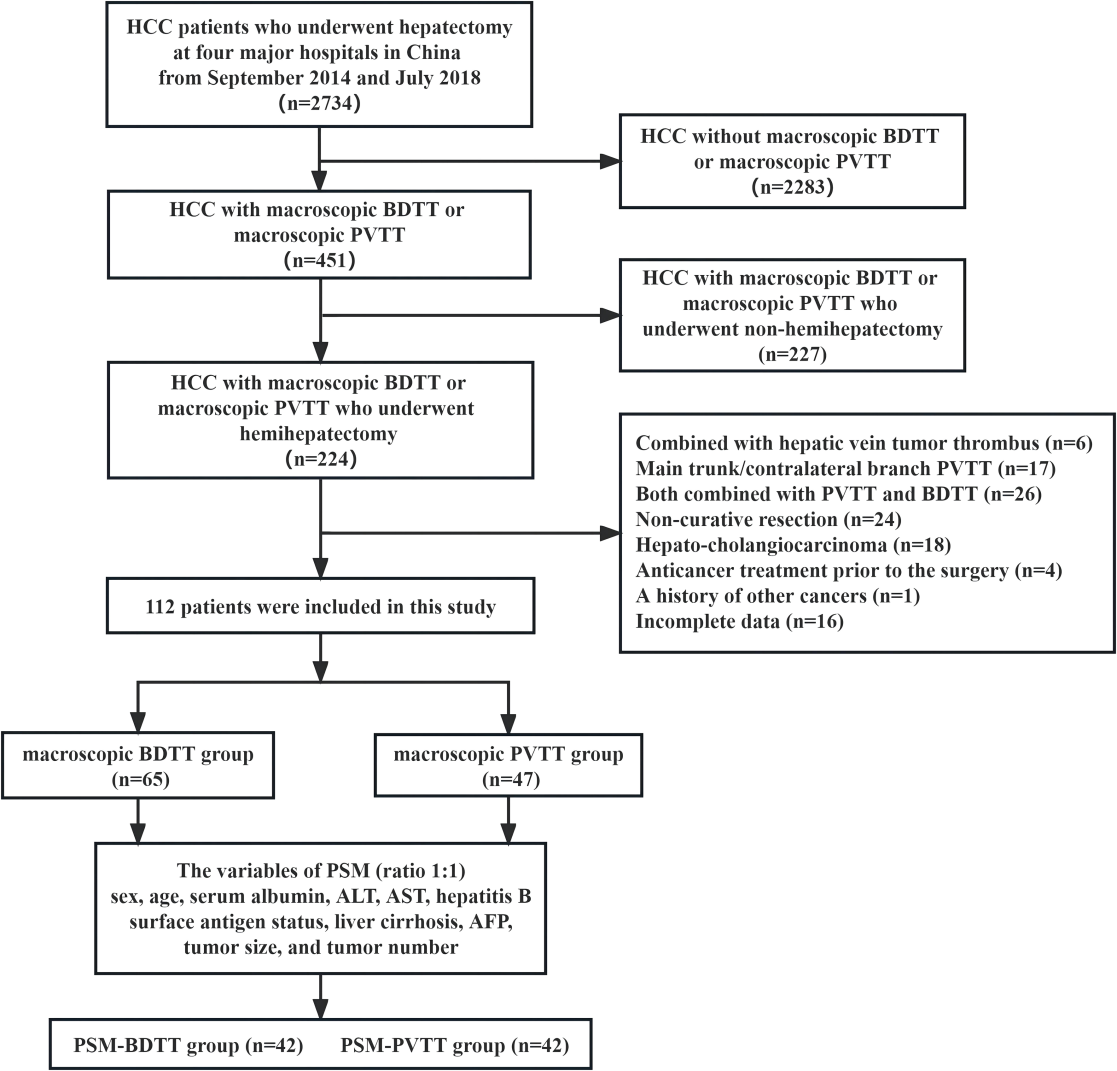
Study design frame, patient selection, and PSM method. HCC, hepatocellular carcinoma; BDTT, bile duct tumor thrombus; PVTT, portal vein tumor thrombus; PSM, propensity score matching; ALT, alanine aminotransferase; AST, aspartate transaminase; AFP, α-fetoprotein.

The inclusion criteria were as follows (1): patients aged 18–75 years with good operative tolerance (2), HCC with macro-BDTT or macro-PVTT confirmed via imaging and histopathological analyses (3), underwent curative hemihepatectomy, and (4) no extrahepatic or distant metastases. The exclusion criteria were as follows (1): combined with hepatic vein tumor thrombus (6 patients) (2), main trunk/contralateral branch PVTT (17 patients) (3), both combined with PVTT and BDTT (26 patients) (4), non-curative resection (24 patients) (5), hepato-cholangiocarcinoma (18 patients) (6), anticancer treatment prior to the surgery (4 patients) (7), history of other cancers (1 patient), and (8) incomplete data (16 patients) (**Figure 1**).

### 2.2 Procedures

All individuals had undergone preoperative serological and imaging examinations. General information (such as sex, age, disease, and treatment history) was obtained from all participants. Additionally, data on other examinations including laboratory tests for liver function (serum albumin, total bilirubin, alanine aminotransferase, and aspartate aminotransferase), tests for hepatitis B virus infection and serum AFP levels, imaging examinations (abdominal ultrasonography, contrast-enhanced MRI or CT), surgical and postoperative therapy, postoperative histopathological examinations was collected. Child-Pugh classification was assessed according to the study by Pugh (31). The albumin bilirubin (ALBI) score and grading system was used for this study as outlined by Johnson (32): three grades were defined based on the following thresholds: ALBI grade 1 (G1) ≤ -2.60, ALBI grade 2 (G2) > -2.60 and ≤ -1.39, and ALBI grade 3 (G3) > -1.39.

Intraoperative ultrasonography was used routinely to determine the tumor margin and transection plane and define the relationship between the tumor and major vasculatures. After ligating the hepatic artery and portal vein individually, the ipsilateral hemihepatic and first branch of the bile duct or portal vein containing the tumor thrombus were resected integrally. For patients with PVTT, the blood inflow of the liver was occluded using Pringle’s maneuver at a site distal to the tumor thrombus. For patients with BDTT, extrahepatic bile duct resection (BDR) was performed if the tumor thrombus was poorly removed intraoperatively or extrahepatic bile duct invasion was detected.

Postoperative days 1, 3, and 5 involved liver function and routine blood tests. The postoperative complications were recorded using the Clavien–Dindo classification (33). The degree of liver cirrhosis, tumor number, tumor size, tumor differentiation, and type of tumor thrombus were determined by pathological examinations. Tumor size was defined as the maximum diameter of the largest tumor in the resected specimen. The histologic grade of tumor differentiation was assigned according to the Edmonson–Steiner classification (34).

### 2.3 Follow-up

Preventive transarterial chemoembolization (TACE) was recommended for all patients 4‒8 weeks after surgery. Follow-up was performed every 3 months for the first year and every 6 months thereafter until death. Further, the following tests were performed: serum AFP level test, liver function test, and contrast-enhanced CT or MRI. If there was confirmation of recurrence during the follow-up visits, the optimal treatment, which could include ablation, surgical resection, TACE, systemic therapy, or a combination of these approaches, was carried out in accordance with the clinical practice guidelines for HCC, multidisciplinary team’s decision for HCC management and the patient’s general condition.

The OS and recurrence-free survival (RFS) rates were the primary and secondary endpoints, respectively, of this study. OS was defined as the period from the date of hepatectomy to the date of death due to any cause or the date of the last follow-up, and RFS was defined as the period from the date of hepatectomy to the date of the first locoregional recurrence, distant metastasis, or last follow-up. All patients were followed-up until death or the conclusion of the study in September 2023.

### 2.4 Statistical analysis

PSM was performed to overcome deviations caused by baseline imbalance. A logistic regression model was used to calculate propensity scores. Propensity scores were calculated on the basis of clinically important variables and factors in relation to survival, including sex, age, serum albumin, alanine aminotransferase, and aspartate aminotransferase levels, hepatitis B surface antigen status, liver cirrhosis, AFP level, tumor size, and tumor number. PSM analyses were performed using the MatchIt R package (version 4.5.5) with a caliper width set equal to 0.2 standard deviations of the logit of the propensity score (35), and a 1:1 nearest-neighbor matching approach between the BDTT and PVTT groups.

Categorical variables were expressed as counts and percentages and compared using the chi-squared test or Fisher’s exact test, as appropriate. To show the degree of adjustment for PSM, we calculated standardized mean differences (SMD) for all variables before and after PSM using the stddiff R package (version 3.1) to show the size of differences in patient characteristics between two groups, where 0.8, 0.5, and 0.2 were considered large, medium, and small differences, respectively (36). The Kaplan‒Meier method was used to calculate the recurrence and survival rates, and the log-rank test was used for comparison. In the propensity-matched cohort, survival curves were compared using a stratified log-rank test by matched pairs, and we calculated hazard ratios (HR) via the Cox proportional hazards model with a robust variance estimator to account for the clustering within matched pairs. Additionally, the prognostic factors for RFS and OS before PSM were analyzed, and the variables with P values < 0.05 in univariate analyses, type of tumor thrombus, and factors significant to prognosis (such as sex, age, and ALBI grade) were further entered into the multivariate Cox proportional hazards model, using the enter method to identify independently significant factors. After PSM, non-matched factors (ALBI grade, Edmonson-Steiner grade, type of hemihepatectomy, and type of tumor thrombus) were separately analyzed by univariate Cox regression proportional hazards model to determine adjusted P values and HR. Statistical significance was set at two-tailed P values < 0.05. All statistical analyses were performed using the R software (version 4.3.1).

## 3 Results

### 3.1 Patient characteristics

A total of 112 HCC patients with macro-BDTT or macro-PVTT who underwent hemihepatectomy at the 4 centers were included. Among them, 101 (90.2%) were males and 11 (9.8%) were females. Ninety-six patients (85.7%) were diagnosed with hepatitis B virus infection, and 62 were diagnosed with cirrhosis; the baseline characteristics of the total study population are presented in **Table 1**. Before PSM, there were 65 patients in the BDTT group and 47 patients in the PVTT group. The BDTT group comprised 12 patients with B3 BDTT and 53 patients with B4 BDTT, of whom 5 underwent extrahepatic BDR. After PSM, 42 patient pairs were selected from each group. The PSM-BDTT group comprised 11 patients with B3 BDTT and 31 with B4 BDTT, of whom 3 underwent extrahepatic BDR. The clinical baseline characteristics between the two groups were more comparable and balanced after PSM, with SMD < 0.4 for all variables (**Table 2**).

**Table 1:** Patient demographics and baseline characteristics.

Variables	Patients (n = 112)
Sex (male/female)	101/11
Age (< 65/≥ 65years)	98/14
Albumin (< 35/≥ 35 g/L)	9/103
Total bilirubin (< 34/≥ 34 μmol/L)	80/32
ALBI (G1/G2-G3)	55/57
ALT (< 40/≥ 40 U/L)	32/80
AST (< 40/≥ 40 U/L)	30/82
Child-Pugh class (A/B)	85/27
HBsAg (negative/positive)	16/96
AFP (<400/≥ 400 ng/mL)	68/44
Liver cirrhosis (negative/positive)	50/62
Tumor number (solitary/multiple)	77/35
Tumor size (< 5/≥ 5cm)	40/72
Type of tumor thrombus (BDTT/PVTT)	65/47
Type of hemihepatectomy (left/right)	67/45
Clavien–Dindo classification of complications (I-IIIa/IIIb-V)	108/4
Edmondson-Steiner grade (I-II/III-IV)	23/89

ALBI, Albumin-Bilirubin; ALT, alanine aminotransferase; AST, aspartate transaminase; HBsAg, hepatitis B surface antigen; AFP, α-fetoprotein; BDTT ,bile duct tumor thrombus; PVTT, portal vein tumor thrombus.

**Table 2:** Perioperative characteristics of BDTT and PVTT groups before and after PSM.

Variables	Before PSM (n=112)				After PSM (n=84)			
	BDTT (n=65)	PVTT (n=47)	p Value	SMD	BDTT (n= 42)	PVTT (n= 42)	p Value	SMD
Sex (male/female)^a^	60/5	41/6	0.373	0.168	38/4	37/5	0.724	0.077
Age (<65/≥65 years)^a^	56/9	42/5	0.612	0.098	37/5	37/5	1.000	0.000
ALbumin (<35/≥35 g/L)^a^	7/58	2/45	0.368	0.249	2/40	2/40	1.000	0.000
ALBI grade (G1/G2-G3)	23/42	32/15	**<0.001**	0.693	21/21	28/14	0.121	0.343
ALT (<40/≥40 IU/L)^a^	14/51	18/29	0.053	0.792	11/31	14/28	0.474	0.209
AST (<40/≥40 IU/L)^a^	17/48	13/34	0.859	0.484	11/31	9/33	0.608	0.106
HBsAg (-/+)^a^	12/53	4/43	0.226	0.294	6/36	3/39	0.480	0.232
Liver cirrhosis (-/+)^a^	32/33	18/29	0.251	0.222	20/22	17/25	0.510	0.144
AFP (<400/≥400 ng/mL)^a^	40/25	28/19	0.834	0.140	28/14	26/16	0.649	0.100
Multiple tumors (no/yes)^a^	47/18	30/17	0.339	0.183	31/11	28/14	0.474	0.157
Tumor size (<5/≥5 cm)^a^	28/37	12/35	0.056	0.376	14/28	11/31	0.474	0.157
Type of hemihepatectomy (left/right)	38/27	29/18	0.730	0.362	27/15	27/15	1.000	0.000
Edmondson-Steiner grade (I-II/III-IV)	15/50	8/39	0.434	0.424	7/35	6/36	0.763	0.250

a Variables in the propensity score.

BDTT, bile duct tumor thrombus; PVTT, portal vein tumor thrombus; PSM, propensity score matching; SMD, Standardized mean differences; ALBI, Albumin-Bilirubin; ALT, alanine aminotransferase; AST, aspartate transaminase; HBsAg, hepatitis B surface antigen; AFP, α-fetoprotein.

With a median follow-up of 31 months (range, 3‒139 months), 80 patients experienced recurrence (71.4%) and 74 died (66.1%). The median RFS and OS in this study were 10.6 months (95% confidence interval [CI], 7‒14 months) and 32 months (95% CI, 24‒40 months), respectively. The total 1-, 3-, and 5-year RFS rates were 44.1%, 32.1%, and 29.5%, respectively, and the total 1-, 3-, and 5-year OS rates were 84.8%, 45.6%, and 33.1%, respectively.

### 3.2 Comparison between HCC with macro-BDTT and those with macro-PVTT

**Figure 2:**
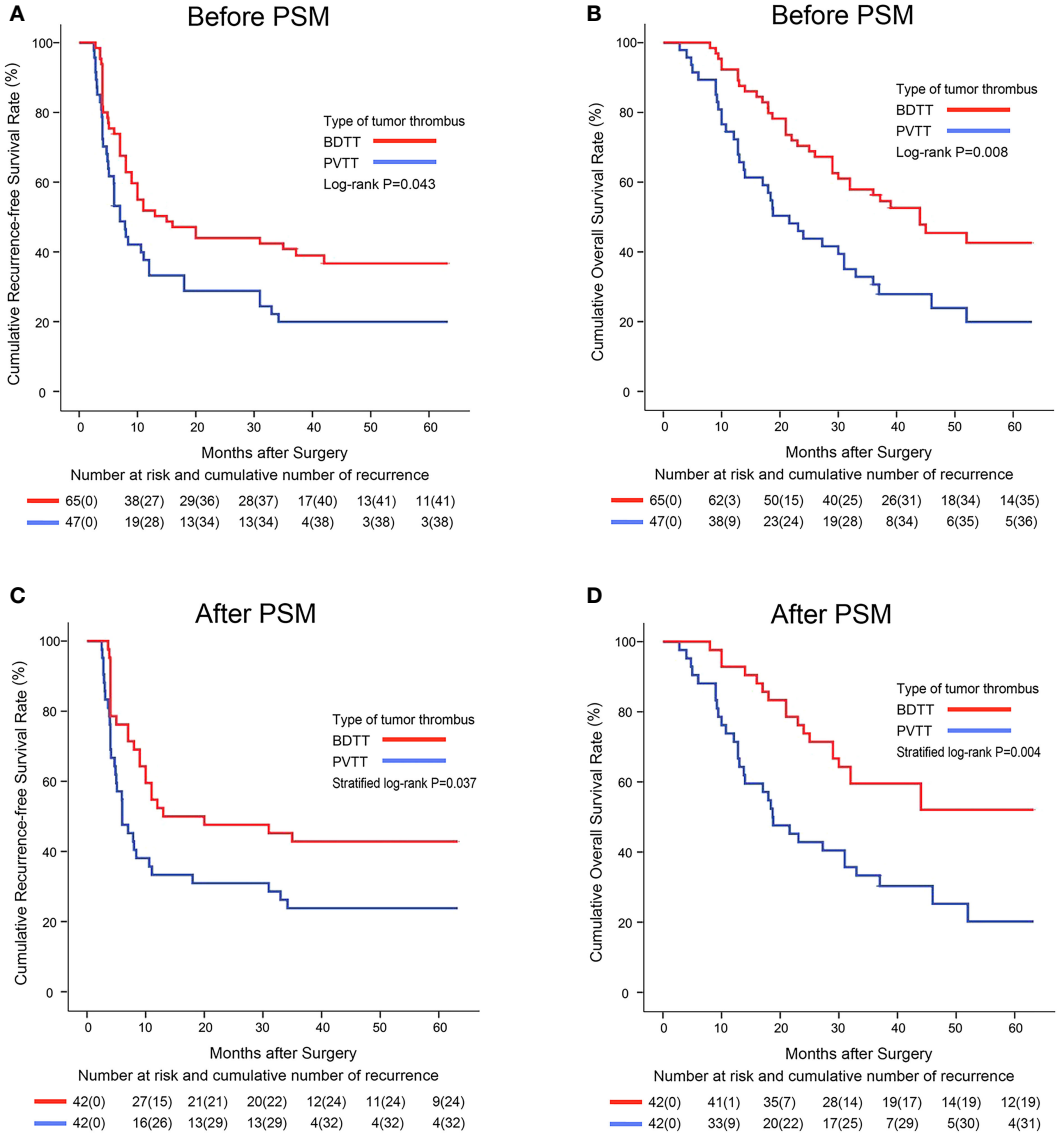
Kaplan–Meier survival curves comparing 5-year recurrence-free and overall survival among hepatocellular carcinoma patients with BDTT or PVTT before **(A, B)** and after PSM **(C, D)**. BDTT, bile duct tumor thrombus; PVTT, portal vein tumor thrombus; PSM, propensity score matching.

Before PSM, the 1-, 3-, and 5-year RFS rates in the BDTT group (51.8%, 40.9%, and 36.7%, respectively) were significantly higher than those in the PVTT group (37.7%, 19.9%, and 19.9%, respectively; P = 0.043, **Figure 2A**). Further, the 1-, 3-, and 5-year OS rates were significantly higher in the BDTT group (92.3%, 56.3%, and 42.6%, respectively) than in the PVTT group (74.5%, 30.7%, and 19.9%, respectively; P = 0.008, **Figure 2B**). There were 38 deaths (38/65, 58.5%) in the BDTT group and 36 (36/47, 76.6%) in the PVTT group.

After PSM, 42 patient pairs were selected. Better RFS and OS were observed in the PSM-BDTT group (P = 0.037 and P = 0.004, respectively). The 1-, 3-, and 5-year RFS rates were 52.4%, 42.9%, and 42.9%, respectively, in the PSM-BDTT group and 33.3%, 23.8%, and 23.8%, respectively, in the PSM-PVTT group. The 1-, 3-, and 5-year OS rates were 92.9%, 59.5%, and 52.1%, respectively, in the PSM-BDTT group and 73.8%, 33.3%, and 20.2%, respectively, in the PSM-PVTT group (**Figures 2C, D**).

### 3.3 Patterns for tumor recurrence

Before PSM, 43 patients (43/65, 66.2%) in the BDTT group and 37 (37/47, 78.7%) in the PVTT group suffered tumor recurrence (P = 0.146). After PSM, 24 patients in the PSM-BDTT group developed tumor recurrence, including 16 with intrahepatic recurrence alone and 8 with extrahepatic recurrence. Thirty-two patients in the PSM-PVTT group developed tumor recurrence, including 28 with intrahepatic recurrence alone and 4 with extrahepatic recurrence. Compared with the PSM-BDTT group, patients in the PSM-PVTT group had a higher likelihood of developing intrahepatic recurrence alone (P = 0.032).

### 3.4 Risk factors influencing RFS and OS

Before PSM, univariate analysis indicated that PVTT and large HCC were risk factors for poor RFS (HR = 1.577; 95% CI, 1.013–2.454, P = 0.043 and HR = 1.668; 95% CI, 1.031–2.697, P =0.037, respectively) and OS (HR = 1.859; 95% CI, 1.174–2.943, P = 0.008 and HR = 1.677; 95% CI, 1.020–2.756, P = 0.042, respectively) (**Table 3**). Multivariate analysis indicated that PVTT (HR = 1.835; 95% CI, 1.121–3.005, P = 0.016) was a risk factor for poor OS, whereas large HCC (HR = 1.553; 95% CI, 1.050–2.536; P = 0.039) was a risk factor for poor RFS (**Table 4**).

**Table 3:** Univariate analysis of factors related to the RFS and OS before PSM.

Variables	RFS			OS		
	HR	(95% CI)	p Value	HR	(95% CI)	p Value
Sex (male)	1.280	0.584-2.807	0.538	1.482	0.673-3.265	0.329
Age (≥ 65 years)	1.464	0.702-3.054	0.309	1.595	0.731-3.478	0.241
Albumin (< 35 g/L)	1.054	0.485-2.291	0.895	1.141	0.523-2.491	0.741
ALBI grade (G2-G3)	1.034	0.666-1.605	0.883	0.946	0.599-1.494	0.810
ALT (≥40 IU/L)	1.349	0.863-2.110	0.189	1.443	0.910-2.287	0.119
AST (≥40 IU/L)	0.978	0.619-1.545	0.924	1.071	0.666-1.723	0.778
AFP (≥400 ng/mL)	1.178	0.752-1.843	0.474	1.158	0.727-1.843	0.537
HBsAg (positive)	1.742	0.975-3.115	0.061	1.593	0.888-2.856	0.118
Liver cirrhosis (positive)	1.052	0.676-1.636	0.824	1.195	0.749-1.907	0.454
Tumor size (≥ 5 cm)	1.668	1.031-2.697	**0.037**	1.677	1.020-2.756	**0.042**
Multiple tumors	1.210	0.765-1.935	0.427	1.196	0.734-1.947	0.473
Edmondson-Steiner grade (III-IV)	1.444	0.914-2.280	0.115	1.505	0.938-2.413	0.090
Type of hemihepatectomy (right)	1.252	0.833-1.896	0.279	1.544	0.826-2.312	0.112
Type of tumor thrombus (PVTT)	1.577	1.013-2.454	**0.043**	1.859	1.174-2.943	**0.008**

Bold values are statistically significant (p < 0.05).

RFS, recurrence-free survival; OS, overall survival; HR, hazard ratio; CI, confidence interval; HBsAg, hepatitis B surface antigen; ALT, alanine aminotransferase; AST, aspartate transaminase; AFP, α-fetoprotein; PVTT, portal vein tumor thrombus.

**Table 4:** Multivariate analysis of factors related to the RFS and OS before PSM.

Variables	RFS			OS		
	HR	(95% CI)	p Value	HR	(95% CI)	p Value
Sex (male)	1.333	0.599-2.963	0.481	1.667	0.746-3.723	0.213
Age (≥ 65 years)	0.734	0.346-1.555	0.419	0.694	0.311-1.547	0.371
ALBI grade (G2-G3)	1.126	0.709-1.787	0.616	1.028	0.629-1.679	0.913
Maximum tumor size (≥ 5 cm)	1.553	1.050-2.536	**0.039**	1.499	0.746-3.723	0.119
Type of tumor thrombus (PVTT)	1.511	0.941-2.427	0.087	1.835	1.121-3.005	**0.016**

Bold values are statistically significant (p < 0.05).

RFS, recurrence-free survival; OS, overall survival; HR ,hazard ratio; CI, confidence interval; PVTT, portal vein tumor thrombus.

After PSM, univariate analysis revealed that PVTT represented a risk factor for both poor RFS (HR = 1.749; 95% CI, 1.034–2.958; P = 0.037) and poor OS (HR = 2.245; 95% CI, 1.286–3.921; P = 0.004) (**Supplementary Table 1**).

## 4 Discussion

PVTT is widely established to have a significantly negative impact on the prognosis of HCC. Because of recent advances in imaging and a better knowledge of BDTT, a growing percentage of HCC patients with BDTT were diagnosed preoperatively. Currently, there are fewer studies on BDTT, and the findings are still controversial with varying conclusions (14–19, 22–24). Yang et al. concluded that the prognosis of HCC patients with BDTT is poorer than that of patients with PVTT (25). However, PVTT is an essential factor in the staging of HCC, whereas BDTT is not a staging factor for HCC (12, 13). Therefore, we conducted this study to provide evidence for the effect of BDTT on the long-term prognosis of HCC. In our study, the 3- and 5-year OS rates of HCC patients with macro-BDTT and those with macro-PVTT after surgery were 56.3% and 42.6%, and 30.7% and 19.9%, respectively. Patients with macro-BDTT had better OS and RFS after surgery than those with macro-PVTT before and after PSM.

HCC is a highly aggressive malignancy in which a tumor thrombus easily invades the Glisson sheath to form a PVTT or BDTT. Over several decades, HCC patients with macro-PVTT or macro-BDTT have generally received conservative treatment with a grave prognosis. Several studies (9, 10, 22–24) have indicated that aggressive hemihepatectomy can improve the prognosis of HCC patients with macro-PVTT or macro-BDTT. Kokudo et al. (9) and Liang et al. (10) have reported that surgical treatment significantly increased OS and RFS in HCC patients with VP1 to VP3 PVTT but not with VP4 PVTT. Surgery is rarely recommended for HCC patients with VP4 PVTT since they typically have a poor prognosis; instead, conservative treatment is administered. Therefore, we excluded those with VP4 PVTT to avoid the impact of VP4 PVTT on our analysis. In contrast, previous studies (18, 24) have reported that the long-term survival of HCC patients with macro-BDTT was satisfactory after curative resection and no statistical difference has been found in the long-term prognosis of HCC patients with B3 BDTT and B4 BDTT (18, 37). Thus, patients with B3 and B4 BDTT were included in the present study.

In our study, OS and RFS were significantly worse in patients with macro-PVTT than in those with macro-BDTT before and after PSM. These findings may be associated with the highly aggressive biological behavior of HCC, tending toward intrahepatic metastasis via the portal vein (38). Zhou et al. (39) have reported that the concentration of thromboregulatory proteins (which can inhibit fibrin synthesis and prevent cancer cells from adhering to portal vein endothelial cells) was lower in the plasma of HCC patients with PVTT than in those without PVTT. Shimizu et al. (40) have proposed that HCC patients with PVTT had significantly higher levels of soluble intercellular adhesion molecule type I (which may enable tumor cells to invade the surrounding tissue and extracellular matrix) in their serum than those in HCC patients without PVTT. Thus, HCC patients with PVTT are prone to intrahepatic recurrence with an extremely poor prognosis, as we also confirmed in this study. Regarding BDTT, since the tumor thrombus grows with an expansive cast shape and the bile flow is slow, it does not easily metastasize along the duct and rarely invades the extrahepatic bile duct (41, 42). Additionally, as we demonstrated previously, BDTT was associated with significantly worse long-term surgical outcomes in HCC patients with the American Joint Committee on Cancer (AJCC) stages I and II, but not in those with AJCC stage III (43). Another study (44) has further indicated that bile duct invasion could be considered an independent prognostic factor for survival in early-stage HCC but not in advanced-stage HCC.

In this study, only HCC patients who had undergone hemihepatectomy were included. In our previous study (24), anatomic resections were recommended for HCC patients with BDTT if feasible. Wong et al. (18) have indicated that major hepatectomy should be the standard treatment for HCC with macro-BDTT. Similarly, Chen et al. (45) have proposed that hepatectomy should be the standard treatment for HCC with macro-PVTT. Anatomic hemihepatectomy has been reported to yield acceptable perioperative and long-term outcomes in selected HCC patients with macro-PVTT or macro-BDTT (24, 41, 45, 46).

The current study has several limitations. First, despite performing PSM in advance, baseline imbalance could not be completely avoided. Second, as a multicenter study, variability and lack of standardization in the operative and perioperative management were inevitable among the institutions, which may have affected patient survival. Third, this study was conducted in China, where HBV infection is prevalent as a cause of HCC. It is necessary to validate these findings using other groups with HCC caused by different etiologies.

## 5 Conclusions

Despite tumor thrombus affecting the prognosis of HCC patients, reasonably acceptable long-term outcomes could be obtained in HCC patients with macro-BDTT after aggressive hemihepatectomy. Furthermore, the long-term surgical outcomes of HCC patients with macro-BDTT were significantly better than those of HCC patients with macro-PVTT.

## Data availability statement

The raw data supporting the conclusions of this article will be made available by the authors, without undue reservation.

## Ethics statement

The studies involving humans were approved by The Institutional Review Board of Fujian Provincial Hospital. The studies were conducted in accordance with the local legislation and institutional requirements. Written informed consent for participation was not required from the participants or the participants’ legal guardians/next of kin in accordance with the national legislation and institutional requirements.

## Author contributions

YL: Conceptualization, Data curation, Writing – original draft, Writing – review & editing. SW: Conceptualization, Data curation, Writing – original draft, Writing – review & editing. YF: Data curation, Writing – original draft. ZZ: Methodology, Writing – review & editing. LH: Methodology, Writing – review & editing. JL: Methodology, Writing – review & editing. WC: Methodology, Writing – review & editing. YW: Data curation, Writing – review & editing. ZZ: Data curation, Writing – review & editing. JZ: Data curation, Writing – review & editing. JW: Conceptualization, Funding acquisition, Resources, Supervision, Writing – review & editing. MY: Conceptualization, Funding acquisition, Project administration, Resources, Supervision, Writing – review & editing.

## Supplementary material

Supplementary Table 1 Univariate analysis of non-matched factors related to the RFS and OS after PSM. Bold values are statistically significant (p < 0.05). RFS, recurrence-free survival; OS, overall survival; HR, hazard ratio; CI, confidence interval; ALBI, Albumin-Bilirubin; PVTT, portal vein tumor thrombus.
