# Supplementary material for: Long-term surgical outcomes of bile duct tumor thrombus versus portal vein tumor thrombus for hepatocellular carcinoma: a propensity score matching analysis
Source: Front Oncol. 2024 Apr 2;14:1372123. doi: 10.3389/fonc.2024.1372123 (PMC11018934; doi:10.3389/fonc.2024.1372123)
Supplement: Supplementary Table 1 — Univariate analysis of non-matched factors related to the RFS and OS after PSM. Bold values are statistically significant (p < 0.05). RFS, recurrence-free survival; OS, overall survival; HR, hazard ratio; CI, confidence interval; ALBI, Albumin-Bilirubin; PVTT, portal vein tumor thrombus. [file Table_1.docx]

| **Supplementary Table 1** Univariate analysis of non-matched factors related to the RFS and OS after PSM | | | | | | | |
| --- | --- | --- | --- | --- | --- | --- | --- |
| Variables | RFS | | |  | OS | | |
|  | HR | (95% CI) | p Value |  | HR | (95% CI) | p Value |
| ALBI grade (G2-G3) | 1.100 | 0.649-1.864 | 0.723 |  | 1.026 | 0.591-1.784 | 0.926 |
| Edmondson-Steiner grade (Ⅲ-Ⅳ) | 0.826 | 0.478-1.429 | 0.495 |  | 0.667 | 0.379-1.175 | 0.161 |
| Type of hemihepatectomy (right) | 1.205 | 0.701-2.070 | 0.500 |  | 1.154 | 0.653-2.041 | 0.622 |
| Type of tumor thrombus (PVTT) | 1.749 | 1.034-2.958 | **0.037** |  | 2.245 | 1.286-3.921 | **0.004** |
| **Notes:** Bold values are statistically significant (p < 0.05)  **Abbreviations:** RFS recurrence-free survival, OS overall survival, HR hazard ratio, CI confidence interval, ALBI Albumin-Bilirubin, PVTT portal vein tumor thrombus | | | | | | | |
